# Perceived stress and harmonious passion: opposing correlates of burnout in entrepreneurs

**DOI:** 10.3389/fpsyg.2026.1922166

**Published:** 2026-10-14

**Authors:** Tibor Dőry, Lili Anna Hujber, Attila Szabo

**Affiliations:** 1 Kautz Gyula Faculty of Business and Economics, Széchenyi István University, Győr, Hungary; 2 Doctoral School of Psychology, Faculty of Education and Psychology, ELTE Eötvös Loránd University, Budapest, Hungary; 3 Faculty of Health and Sport Sciences, Széchenyi István University, Győr, Hungary

**Keywords:** entrepreneurial burnout, job demands-resources model, passion, personal resources, stress, work addiction

## Abstract

**Purpose:**

This study examines how harmonious and obsessive passion, perceived stress, and work addiction relate to burnout in entrepreneurs, using the Job Demands-Resources (JD-R) model, extended to personal resources, as the organizing framework.

**Methods:**

A cross-sectional online study was conducted with 184 Hungarian entrepreneurs (23.4% women; mean age = 54.30 years, SD = 10.76). Participants completed validated Hungarian versions of the Mini Oldenburg Burnout Inventory, Passion Scale, Perceived Stress Scale, and Bergen Work Addiction Scale. After a confirmatory factor analysis of the burnout measure, a hierarchical regression entered all four focal variables together, while controlling for age, gender, and weekly working hours, and tested whether harmonious passion moderated the association between stress and burnout.

**Results:**

The four variables explained an additional 54% of the variance in burnout (total *R*^2^ = 0.57). Harmonious passion (*β* = −0.39) and perceived stress (*β* = 0.38) had the strongest unique associations, in opposite directions. Work addiction showed a smaller positive association (*β* = 0.17) that was confined to exhaustion. Obsessive passion was not uniquely related to burnout, and harmonious passion did not buffer the association between stress and burnout.

**Conclusion:**

Perceived stress and harmonious passion are strong, opposing correlates of burnout in entrepreneurs, and compulsive working adds a smaller, exhaustion-specific risk. Harmonious passion behaved as an additive personal resource rather than as a buffer against stress. Because the design was cross-sectional, longitudinal studies are needed before causal conclusions can be drawn. Supporting self-chosen, flexible engagement with work and paying attention to compulsive work patterns are promising targets for programs that aim to protect entrepreneurs’ wellbeing.

## 1 Introduction

Entrepreneurship is a continuous roller coaster ride that offers opportunities for both positive and negative wellbeing (Stephan et al., 2023). On the one hand, building a business brings joy, pride, and satisfaction to the owner. On the other hand, long working hours, constant problem-solving, and the need for continuous readiness can lead to anxiety and burnout among entrepreneurs (Wiklund et al., 2019). Unlike other professions, entrepreneurship is deeply tied to personal identity, as it embodies autonomy, independence, and risk-taking, which best reflect an entrepreneur’s personality (Rauch and Frese, 2007). This unique perception of entrepreneurial work extends beyond the entrepreneur, influencing both their family and broader social environment (Stephan, 2018). Negative wellbeing has a significant effect on the performance of the business and the wellbeing of the employees, with a substantial impact on society as a whole (Torrès and Thurik, 2019).

Entrepreneurial burnout is frequently discussed in academic literature, but Ross et al. (2020) emphasize that only a limited number of researchers have been able to address the core questions of this issue. Burnout among entrepreneurs and managers is a significant workplace concern, as it can harm individual wellbeing and organizational productivity. Lechat and Torrès (2017) note that there have been few empirical studies on the health of small business owners in the last 20 years, and that their results are contradictory. They also point out that empirical research on business owners’ occupational determinants of health is still in its early stages. Torrès and Thurik (2019) go even further, arguing that despite the social and economic importance of small businesses, the study of the health of entrepreneurs is absent from the literature. Palmer et al.'s (2021) comprehensive literature review also confirms that burnout is a rarely discussed topic in entrepreneurship research and suggests that it should be investigated in the reality of its multidimensional professional context.

Burnout is a psychological condition marked by emotional exhaustion, detachment, and a sense of reduced professional efficacy (Schaufeli et al., 1996). Exhaustion reflects depleted energy, cynicism involves a detached and negative attitude toward work, and inefficacy denotes feelings of incompetence and difficulty managing work challenges (Schaufeli et al., 1996; Schaufeli et al., 2002). Burnout negatively impacts both personal and social functioning, leading to a decline in the quality of work. As a result, it affects not only the individual experiencing burnout but also everyone connected to that person (Maslach and Leiter, 2016). Initially studied in human service professions, burnout is now recognized as a prevalent outcome of prolonged work-related stress across diverse occupations, job positions, and among students (e.g., Cooper et al., 2001; Schaufeli et al., 2002). A high risk of burnout is characterized by a low harmonious passion for work (Fernet et al., 2014), while a positive association exists between obsessive passion and burnout (Fernet et al., 2014). The latter could be associated with work addiction. Researchers have started broadening their focus on the factors contributing to burnout (Maslach and Leiter, 2016). The job-demands-resources model (JD-R) suggests that burnout arises when individuals are confronted with job demands and lack the necessary resources to manage those demands (Bakker and Demerouti, 2007). To our knowledge, however, no study has examined passion, perceived stress, and work addiction together as correlates of burnout in entrepreneurs.

There are good reasons to study these variables together rather than one at a time, because they overlap both conceptually and empirically. Obsessive passion and work addiction both describe a compulsive, hard-to-control involvement with work (Vallerand et al., 2003; Andreassen, 2014), and both tend to co-occur with feeling overwhelmed by demands, which the Perceived Stress Scale can capture (Cohen et al., 1983). When each variable is examined on its own, it is impossible to tell whether obsessive passion carries a risk of its own or merely reflects compulsive working and high strain, or whether harmonious passion is linked to lower burnout beyond simply going together with less stress. A joint model can answer these questions. This matters particularly for entrepreneurs. Compared with employees, business owners enjoy considerable autonomy and control over their work (Hessels et al., 2017; Stephan, 2018), conditions that should favor a harmonious form of engagement. At the same time, they carry personal financial risk, work under role overload and ambiguity, and often find it hard to separate work from private life (Stephan, 2018; Wiklund et al., 2019), conditions in which work can easily become compulsive. Which of these forces dominates the burnout picture in this occupational group is still unclear. The only study we found that looked at both forms of passion in relation to entrepreneurial burnout (De Mol et al., 2018) did not consider perceived stress or work addiction, and it did not test whether harmonious passion weakens the link between stress and burnout. We address these gaps in a sample of established Hungarian business owners, a Central and Eastern European group that is rarely represented in this literature.

### 1.1 The connection between passion and burnout

Passion for work reflects a deep affinity for and commitment to an activity that one finds meaningful and enjoyable (Vallerand et al., 2003). The widely popular dualistic model of passion distinguishes between two contrasting forms: harmonious and obsessive passion. Vallerand (2008) suggests that passion arises when individuals love an activity, choose to engage in it freely, and integrate it into their identity. Harmonious passion (HP) occurs when the activity is attractive and naturally internalized, allowing for flexible engagement, which fosters positive emotions and minimizes negative ones (Stenseng et al., 2011; Vallerand et al., 2003, 2006; Vallerand and Miquelon, 2007). In contrast, obsessive passion (OP) is driven by a controlled internalization, leading to rigid, obligatory behavior related to negative emotions and reliance on the activity for self-esteem or problem avoidance (Vallerand, 2010).

Several studies have examined the relationship between passion and burnout across different work environments. Castillo et al. (2017) studied 161 physical education teachers in Spain, finding that HP negatively predicted burnout, while OP was a positive predictor. Similarly, Shah et al. (2020) observed a positive correlation between OP and burnout among 81 special education teachers in Pakistan. Fernet et al. (2014) reported that HP negatively predicted the risk of burnout among novice Canadian teachers, while OP positively correlated with emotional exhaustion and depersonalization. Benitez et al. (2023) studied 748 frontline employees in Spain and found that HP weakened the negative relationship between burnout and intrinsic job satisfaction; accordingly, they described HP as a personal resource within the JD-R model. The same pattern has been observed among entrepreneurs. In a sample of 326 individuals working in entrepreneurial jobs, De Mol et al. (2018) found that HP was negatively while OP was positively related to burnout. Furthermore, Stroe et al. (2020) showed that HP dampened the link between entrepreneurs’ fear of failure and negative affect.

Studies by Saville et al. (2018) and Vallerand et al. (2010) also linked HP with reduced burnout and greater satisfaction across student and nurse populations, emphasizing its protective role against emotional exhaustion and work conflict. Horwood et al. (2021) examined school principals, and their findings suggest that their work passion could help prevent burnout, but only if the passion manifested harmoniously.

Despite increased interest in entrepreneurial burnout and a growing number of empirical studies, little research has been published in this area, leaving unanswered questions about the relationship between entrepreneurship and health. Apart from De Mol et al. (2018), few studies have examined HP and OP in relation to entrepreneurs’ burnout, and none, to our knowledge, has done so alongside stress and work addiction. Based on past work, we expected OP to be positively and HP negatively related to entrepreneurs’ burnout. Research on athletic burnout has also shown that HP and stress relate to burnout in opposite directions (Moen et al., 2016).

### 1.2 Stress and burnout

Research reveals a significant link between job stress and burnout in managers, impacting health, wellbeing, commitment, and performance. For example, Jamal and Baba (2000) found that job stress strongly correlated with burnout dimensions (e.g., emotional exhaustion) and job dissatisfaction. Furthermore, a meta-analytic review by Membrive-Jiménez et al. (2020) found high emotional exhaustion among nursing managers, driven by work overload and inadequate support, with a burnout prevalence of 29%. Johnson et al. (2013) showed that age influences stress responses, and older employees better use coping strategies to mitigate burnout. Lubbadeh (2020) expanded burnout research to non-social service professions, identifying key dimensions and interventions, and concluded that job burnout is a stress-induced condition that harms employees, managers, entrepreneurs, and their organizations. Finally, Wong and Spence Laschinger (2015) emphasized that job stress could predict burnout, lower commitment, and higher turnover in healthcare managers, stressing the need for balanced role demands. Among small business owners, stressful work events have likewise been linked to poorer health (Lechat and Torrès, 2017). These findings justify studying stress and burnout in entrepreneurs to enhance wellbeing and organizational stability.

### 1.3 Work addiction and burnout

Work addiction is a synonym for workaholism, reflecting the inability to detach from work despite negative consequences on health, personal, and social life (Andreassen, 2014). Studies consistently show the detrimental effects of work addiction on burnout, emphasizing the need to understand the mechanism of their relationship. For example, Moyer et al. (2017) found that workaholism negatively impacts psychological capital (PsyCap), increasing the risk of burnout through emotional exhaustion, depersonalization, and reduced personal accomplishment. Similarly, a study of Brazilian nursing science professors found that excessive and compulsive work strongly correlates with higher emotional exhaustion and depersonalization (de Almeida et al., 2020). Furthermore, Nonnis et al. (2018) showed that excessive work, a dimension of workaholism, predicts burnout and disillusion among nurses. Additionally, Bereznowski et al. (2023) unveiled a direct relationship between work addiction symptoms and burnout, identifying mood modification and withdrawal as key pathways to exhaustion. Finally, Towch et al. (2024) further emphasized the association between work addiction and burnout, with PsyCap and job satisfaction partially mediating the relationship. Work addiction also overlaps with OP, since both involve an inner compulsion to work, and OP is closely related to workaholism (Birkeland and Buch, 2015). Whether entrepreneurs are especially prone to such patterns is unclear. With the instrument used in the present study, 8.3% of a representative sample of Norwegian employees met the criterion for work addiction (Andreassen et al., 2014), but comparable figures for business owners are scarce. Despite the lack of such research, past empirical findings and theory provide a strong rationale for studying the association of work addiction with entrepreneurial burnout along with stress and passion, which has not been investigated to date.

### 1.4 Theoretical framework and hypotheses

We used the JD-R model (Demerouti et al., 2001; Bakker and Demerouti, 2017) as the organizing framework. The model distinguishes a health-impairment pathway, in which demands that require sustained effort deplete energy and lead to exhaustion, from a motivational pathway, in which resources foster engagement (Bakker et al., 2014). It also proposes that resources can buffer the effect of demands on strain (Bakker and Demerouti, 2007). Later work extended the model to personal resources, that is, aspects of the self that are linked to resilience and to a sense of control over one’s environment (Xanthopoulou et al., 2007). Because none of our variables is a job characteristic in the strict sense, we position them as follows.

Perceived stress is not a job demand but a person’s appraisal that current demands exceed his or her ability to cope (Cohen et al., 1983; Lazarus and Folkman, 1984). We therefore treat it as a strain-related indicator of how heavy demands are experienced, located on the health-impairment pathway, rather than as a demand in its own right. OP and work addiction are individual work orientations and behavioral patterns, not features of the job. Both describe a compulsive, poorly regulated involvement with work (Vallerand et al., 2003; Andreassen et al., 2012) that can raise the demands people impose on themselves and limit recovery; the updated JD-R theory acknowledges such self-generated demands under the heading of self-undermining (Bakker and Demerouti, 2017). We therefore regard OP and work addiction as person-level risk factors. HP, in turn, fits the definition of a personal resource: it reflects an autonomously internalized, flexible commitment to work that supports engagement while leaving room for other life domains (Vallerand et al., 2003; Benitez et al., 2023).

Conservation of Resources (COR) theory (Hobfoll, 1989; Hobfoll et al., 2018) offers a complementary account. It holds that strain follows the actual or threatened loss of valued resources, and that people with more resources are less vulnerable to such losses. From this perspective, HP and perceived stress may relate to burnout additively, as a resource and as a loss-related appraisal, respectively, even if HP does not change the strength of the link between stress and burnout. A direct test of the JD-R buffering hypothesis therefore helps to decide which account fits the data better.

On this basis, we tested the model shown in Figure 1 and the following hypotheses:

**Figure 1:**
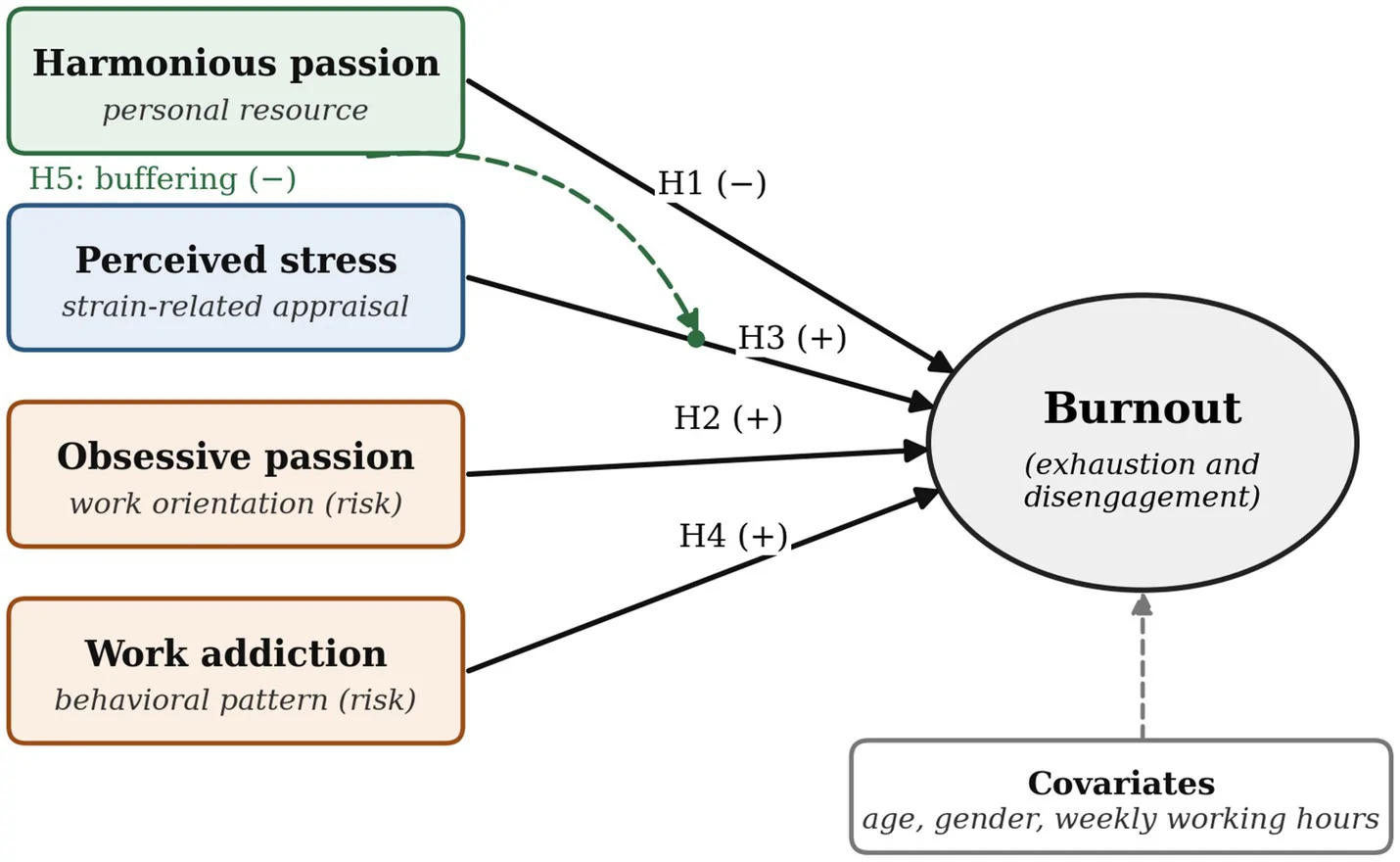
Hypothesized model. Harmonious passion is positioned as a personal resource, obsessive passion and work addiction as person-level risk factors, and perceived stress as a strain-related appraisal of demands. H1–H4 denote the expected associations with burnout, and H5 the expected buffering role of harmonious passion. Age, gender, and weekly working hours were included as covariates. This model is AI-generated based on the authors’ prompt. The authors endorse the model.

*H1*. Harmonious passion is negatively associated with burnout.

*H2*. Obsessive passion is positively associated with burnout.

*H3*. Perceived stress is positively associated with burnout.

*H4*. Work addiction is positively associated with burnout.

*H5*. Harmonious passion moderates the association between perceived stress and burnout, such that the association is weaker at higher levels of harmonious passion.

H1–H4 were tested simultaneously, controlling for age, gender, and weekly working hours. H5 corresponds to the buffering hypothesis of the JD-R model.

## 2 Methodology

### 2.1 Instruments

This survey consisted of demographic questions (e.g., age, gender, education level, family status, number of children), management-related questions (e.g., geographical location of the business, job position, experience), and four psychometric instruments described below.

#### 2.1.1 Mini Oldenburg Burnout Inventory

Burnout was measured using the Mini Oldenburg Burnout Inventory (MOLBI; Thun et al., 2014), a 10-item short form of the Oldenburg Burnout Inventory (OLBI; Demerouti et al., 2003). In this study, the Hungarian version was used (Ádám et al., 2020; Mészáros et al., 2020). The questionnaire can be applied as a measurement for any profession to evaluate the level of occupational burnout. It consists of two dimensions: Exhaustion and Disengagement. Exhaustion measures work-related fatigue and emotional, cognitive, and physical strain of work (e.g., “There are days when I feel tired before I arrive at work”). Disengagement assesses loss of interest in work, depersonalization, loss of commitment, and cynicism (e.g., “It happens more and more often that I talk about my work in a negative way”). The inventory includes ten items, five for each subscale, of which two per subscale are reverse-keyed. Respondents rate the statements on a 4-point scale (1 = totally disagree, 4 = totally agree); after reverse-scoring, higher scores indicate more burnout (Mészáros et al., 2020). In studies of the full 16-item OLBI, alphas have typically ranged from 0.74 to 0.87 for Exhaustion and from 0.70 to 0.83 for Disengagement (Demerouti et al., 2003; Halbesleben and Demerouti, 2005). In the Hungarian adaptation, the Cronbach’s alphas were 0.79 (Exhaustion) and 0.76 (Disengagement; Ádám et al., 2020), and a later Hungarian study supported the use of a global burnout score alongside the two subscales (Mészáros et al., 2020). The psychometric properties of the MOLBI in the present sample, including the treatment of one malfunctioning item, are reported in Section 3.1.

#### 2.1.2 Perceived Stress Scale

Stress levels were assessed using the shortened 4-item version of the Perceived Stress Scale (PSS-4, Cohen et al., 1983). In this study, the Hungarian adaptation was used (Stauder and Konkolÿ Thege, 2006). The 4-item version briefly measures stress perceptions (e.g., “In the last month, how often have you felt difficulties were piling up so high that you could not overcome them?”). The respondents rated the statements on a 5-point Likert scale (0-never, 4-very often). The two positively worded items are reverse-scored, so that higher scores indicate higher perceived stress (range 0–16). In the original validation, the 14-item PSS showed alphas of 0.84–0.86, and the four-item version was also found adequate (Cohen et al., 1983); in the Hungarian adaptation, the PSS-4 had an alpha of 0.79 (Stauder and Konkolÿ Thege, 2006). In our study, Cronbach’s *α* was 0.78 and McDonald’s *ω* was 0.80.

#### 2.1.3 Passion Scale

The Passion Scale (PS, Marsh et al., 2013) was used to measure harmonious passion (HP) and obsessive passion (OP). In the current study, the Hungarian adaptation was used (Tóth-Király et al., 2017). The scale includes 17 statements, with HP and OP each measured on 6-item subscales (e.g., “This activity (work) is in harmony with the other activities in my life” (HP), “If I could, I would only do my activity (work)” (OP)). Five items measure criterion passion. Respondents rated the statements on a 7-point Likert scale (1 do not agree at all, to 7 strongly agree). For scoring, responses were recoded to 0–6 and summed, so that both subscale scores can range from 0 to 36. Both the English and Hungarian versions demonstrated excellent psychometric properties. In the original study, the Cronbach alpha was 0.83 (HP) and 0.86 (OP) (Marsh et al., 2013); in the Hungarian adaptation, it was 0.82–0.86 (HP) and 0.82–0.85 (OP) (Tóth-Király et al., 2017). In the present study, the Cronbach alphas were 0.81 (HP; *ω* = 0.81) and 0.77 (OP; *ω* = 0.77).

#### 2.1.4 Bergen Work Addiction Scale

Work addiction was measured using the Bergen Work Addiction Scale (BWAS, Andreassen et al., 2012). In the present study, the Hungarian version was used (Orosz et al., 2016). This scale consists of seven items about the symptoms of work addiction (e.g., “How often during the last year have you worked to reduce feelings of guilt, anxiety, helplessness, and depression?”). The scale uses a 5-point Likert scale (1-never, 5-always). Item scores were summed (range 7–35), with higher scores indicating more symptoms of work addiction. For descriptive purposes only, respondents who rated at least four of the seven items as “often” (4) or “always” (5) were classified as at risk of work addiction (Andreassen et al., 2012). The Cronbach’s alphas were between 0.80 and 0.84 in the original version (Andreassen et al., 2012) and 0.76 in the Hungarian adaptation (Orosz et al., 2016). The internal reliability in this study was *α* = 0.78 (*ω* = 0.80).

### 2.2 Procedure

This study used the LimeSurvey online data collection platform (LimeSurvey Project Team, 2024) between August and October 2024. The research design was cross-sectional. Data collection was anonymous and based on questionnaires completed by volunteers. Participation was open to individuals aged 18 and older who indicated being wholly or partly owner of a company and working in owner-manager positions, as the call and the consent form specified. The collected data were exported in SPSS (Statistical Package for Social Sciences) file format (IBM SPSS Statistics, Version 28.0; IBM Corp., 2021); the software used for the analyses is described in Section 2.6. Based on sample size calculations (detailed in the following section), data collection ceased after receiving 200 responses. Fifteen responses were excluded because of very short response times or incomplete responses, and one response was excluded due to an unusual response pattern identified through standard residual indices in burnout scores.

### 2.3 Sample size calculation

We determined the minimum required sample sizes for the statistical tests using G*Power (v. 3.1.9.7) software (Faul et al., 2007). We aimed to obtain more than the recommended minimum power in behavioral sciences research (i.e., 1 − *β* = 0.80; Cohen, 1988), and therefore, in calculating the minimum sample sizes, we used a higher power level (1 − *β* = 0.90). First, for analyses of variance (ANOVA; to examine gender differences), we targeted a medium effect size (*f* = 0.25) with *α* = 0.05 and power (1 − *β*) = 0.90. This calculation indicated a minimum required sample size of 172. Second, for two-tailed correlations, we used the following parameters: expected correlation (*ρ*) = 0.30, *α* = 0.05, 1 − *β* = 0.90, and null hypothesis *ρ* = 0.00. The results yielded a required sample size of at least 112. Third, for multiple regression, assuming a fixed model with three predictors, medium effect size (*f^2^* = 0.15), *α* = 0.05, and 1 − *β* = 0.90, the minimum required sample size was 99. Because the final regression model contained seven predictors (three covariates and four focal variables), we repeated this calculation with the same parameters, which indicated a minimum of 130 participants. Our sample size (*n* = 184; 179 with complete covariate data) satisfies the minimum requirement for all tests. For the interaction term (H5), a sensitivity analysis showed that, with *N* = 179, *α* = 0.05, and 1 − *β* = 0.80, the smallest detectable effect was *f^2^* = 0.045, which corresponds to an increase in *R*^2^ of about 0.02 in our model. Smaller interaction effects, which are common in field studies (McClelland and Judd, 1993), could have gone undetected.

### 2.4 Participants

Participants were recruited with the help of the Hungarian Chamber of Commerce and Industry and the National Association of Entrepreneurs and Employers, who shared the call for research participation with their members. Also, we sent about 3,000 direct emails to heads of Hungarian industrial companies with revenues of more than 200,000 euros in 2023. Participation was voluntary, anonymous, and conditional upon giving consent to participate by selecting the “yes” button at the bottom of the online consent form. The sample comprised 184 entrepreneurs, including 43 females and 140 males, and one participant did not disclose gender. Their mean age was 54.30 (SD = 10.76), ranging between 25 and 82. Of the 184 participants, 124 (67.4%) were executive managers of their companies, 27 (14.7%) were top managers, and 33 (17.9%) were co-owners of the company. Most respondents (141 or 76.6%) had either college, university, or postgraduate education, while 43 (23.4%) had completed secondary or high school. All participants were Hungarian citizens. Most had a partner (148 cohabiting, 16 living separately), while 20 were single. Most (131) had two or more children, 34 had one child, and 19 had no children. Participants reported having an average of 23.13 (SD = 10.73; *n* = 170) years of managerial experience. Most (100) respondents were from county capitals, 51 were from other Hungarian cities, and 33 were from rural areas.

The sample consisted of participants with an average of 20.94 years of entrepreneurship experience (SD = 11.11; *n* = 170), ranging from less than 1 year to 43 years. The percentage ownership of their companies ranged from 1 to 100%, with an average of 65.72% (SD = 32.59%). The number of companies fully owned by the participants ranged from zero to five, with an average of 1.76 companies (SD = 1.09). Finally, participants reported working in their companies an average of 46.13 h per week (SD = 17.57; *n* = 180), ranging from one to 100 h.

### 2.5 Ethics statement

The Ethics Committee of the Széchenyi István University granted ethical permission for this study [SZE/ETT-3/2024 (V.26)] and assured that the study followed the ethical principles outlined in the Declaration of Helsinki (World Medical Association, 2013).

### 2.6 Data analysis

The analyses followed four steps. First, we examined the factor structure of the MOLBI with confirmatory factor analysis (CFA), comparing a one-factor model with a model of two correlated factors (Exhaustion and Disengagement). Because the items have four response categories, we used the WLSMV estimator for ordinal indicators (Li, 2016) and evaluated fit with CFI and TLI (≥0.95), RMSEA (≤0.08), and SRMR (≤0.08) as guidelines (Hu and Bentler, 1999). Internal consistency was estimated with Cronbach’s *α* and McDonald’s *ω* (McDonald, 1999).

Second, we computed descriptive statistics and Pearson correlations for all study variables and covariates. Third, we tested H1–H5 with a three-step hierarchical multiple regression. The covariates entered in Step 1 were chosen on theoretical grounds rather than on the basis of bivariate significance (Becker et al., 2016): age, because burnout tends to decrease with age and experience (Brewer and Shapard, 2004); gender, because women and men tend to differ in some components of burnout (Purvanova and Muros, 2010); and weekly working hours, as an indicator of quantitative workload that could otherwise confound the associations of work addiction and OP with burnout. Entrepreneurial and managerial experience were not included because they correlated strongly with age (*r* = 0.59 and *r* = 0.64) and were missing for 14 respondents; a model including entrepreneurial experience and firm size is reported as a robustness check. In Step 2, HP, OP, perceived stress, and work addiction were entered simultaneously, as specified in the hypothesized model. In Step 3, the product of mean-centered HP and perceived stress was added to test H5 (Aiken and West, 1991). Work addiction was analyzed as a continuous score, because dichotomizing a continuous measure discards information and reduces statistical power (MacCallum et al., 2002). Five participants with missing covariate data were excluded listwise, leaving *N* = 179 for the regression models. We checked the normality of the residuals, homoscedasticity, influential cases, and multicollinearity, and we report heteroscedasticity-consistent (HC3) tests (Hayes and Cai, 2007) and percentile bootstrap confidence intervals (5,000 resamples) as additional checks. The relative importance of the predictors was estimated with general dominance weights (Azen and Budescu, 2003). We use the term *predictor* in its statistical sense only; given the cross-sectional design, all coefficients describe associations.

Fourth, we assessed the robustness of the results by (a) using the original 10-item burnout score, (b) omitting the covariates, (c) adding entrepreneurial experience and firm size (log-transformed number of employees), (d) analyzing Exhaustion and Disengagement separately, and (e) operationalizing work addiction as the number of symptoms endorsed or as the cut-off classification. The analyses were conducted in R 4.3.3 (R Core Team, 2024), using lavaan 0.6-17 (Rosseel, 2012) for the CFA and psych 2.4.1 (Revelle, 2024) for the reliability estimates. All tests were two-tailed with *α* = 0.05.

## 3 Results

### 3.1 Measurement properties of the burnout scale

Item analysis showed that MOLBI item 10, a reverse-keyed Disengagement item related to work engagement, correlated negatively with the other nine items (corrected item–total *r* = −0.27). In the CFA, it loaded negatively on its intended factor (standardized *λ* = −0.35). Disengagement had *α* = 0.56 with this item and *α* = 0.74 without it. With all ten items, the two-factor model fit better than the one-factor model, but the fit was marginal (Table 1). After item 10 was removed, the two-factor model fit adequately (CFI = 0.973, TLI = 0.962, SRMR = 0.059), although the RMSEA (0.088, 90% CI [0.061, 0.116]) exceeded the conventional cut-off, as is common in models with few degrees of freedom (Kenny et al., 2015). Standardized loadings ranged from 0.57 to 0.87. The two factors were strongly correlated (*r* = 0.80, 95% CI [0.70, 0.89]), close to the value of 0.81 reported by Ádám et al. (2020). Given this overlap and earlier Hungarian evidence that the MOLBI total score is a reliable measure of global burnout (Mészáros et al., 2020), we used the 9-item total score as the main outcome (*α* = 0.85, *ω* = 0.85) and analyzed Exhaustion and Disengagement separately as a supplementary step. The 9-item score correlated at 0.99 with the original 10-item score (*α* = 0.79), and all main analyses were repeated with the latter. The other scales had adequate reliability (*α* = 0.77–0.81; *ω* = 0.77–0.80; Table 2).

**Table 1:** Fit of alternative measurement models for the Mini Oldenburg Burnout Inventory (*N* = 184).

Model	*χ* ^2^	*df*	CFI	TLI	RMSEA [90% CI]	SRMR	Δ*χ*^2^ (Δ*df*)
Ten items							
M1: One factor	116.69^***^	35	0.941	0.924	0.113 [0.091, 0.136]	0.082	–
M2: Two correlated factors	94.14^***^	34	0.957	0.942	0.098 [0.075, 0.122]	0.073	15.14 (1)^***a^
Nine items (item 10 removed)							
M3: One factor	96.96^***^	27	0.948	0.931	0.119 [0.094, 0.145]	0.077	–
M4: Two correlated factors	63.11^***^	26	0.973	0.962	0.088 [0.061, 0.116]	0.059	16.74 (1)^***b^

Estimator: WLSMV (mean- and variance-adjusted weighted least squares for ordinal indicators); *χ*^2^ values are scaled. CFI = comparative fit index; TLI = Tucker–Lewis index; RMSEA = root mean square error of approximation; SRMR = standardized root mean square residual. ^a^M2 vs. M1; ^b^M4 vs. M3 (scaled difference tests). In M2, item 10 loaded −0.35 on Disengagement. In M4, standardized loadings ranged from 0.57 to 0.87, and the factor correlation was 0.80 (95% CI [0.70, 0.89]); with robust maximum likelihood, M4 fitted similarly (*χ*^2^(26) = 43.19, *p* = 0.018; CFI = 0.962; TLI = 0.947; RMSEA = 0.065; SRMR = 0.053). ^***^p < 0.001.

**Table 2:** Descriptive statistics and internal consistency of the study variables (*N* = 184).

Variable	*k*	Possible range	Observed range	*M*	SD	Skewness	Kurtosis	*α*	*ω*
Burnout (MOLBI total)^a^	9	9–36	9–31	18.95	4.98	0.17	−0.64	0.85	0.85
Exhaustion	5	5–20	5–20	11.76	3.23	0.09	−0.49	0.80	0.81
Disengagement^a^	4	4–16	4–13	7.18	2.30	0.42	−0.81	0.74	0.76
Harmonious passion	6	0–36	8–36	24.09	6.07	−0.25	−0.55	0.81	0.81
Obsessive passion	6	0–36	0–31	11.75	6.29	0.19	−0.31	0.77	0.77
Perceived stress (PSS-4)	4	0–16	0–15	5.14	2.75	0.35	0.46	0.78	0.80
Work addiction (BWAS)	7	7–35	9–30	20.01	4.63	−0.04	−0.66	0.78	0.80
Age (years)	–	–	25–82	54.30	10.76	−0.03	−0.03	–	–
Weekly working hours^b^	–	–	1–100	46.13	17.57	0.00	0.92	–	–

*k* = number of items; *α* = Cronbach’s alpha; *ω* = McDonald’s omega; MOLBI = Mini Oldenburg Burnout Inventory; PSS-4 = Perceived Stress Scale, four-item version; BWAS = Bergen Work Addiction Scale. Passion items were scored 0–6. ^a^Without item 10 (see Section 3.1); with all ten items, burnout *M* = 21.37, SD = 4.82, *α* = 0.79, *ω* = 0.82, and Disengagement *α* = 0.56. ^b^*n* = 180. Gender: 43 women, 140 men, 1 not reported.

### 3.2 Descriptive statistics, work addiction, and gender comparisons

Table 2 presents the descriptive statistics. Skewness and kurtosis values of all study variables were between −1 and +1, well within the usual limits for approximate normality (|skewness| < 2, |kurtosis| < 7; Byrne, 2010; Hair et al., 2010).

Based on the Bergen Work Addiction Scale’s (BWAS) cut-off score for workaholics (Andreassen et al., 2012), which proposes that a score of “often” or “always” on at least 4 out of the 7 BWAS items can reflect work addiction, we created two groups: (1) not addicted (*n* = 128, 69.6%) and (2) addicted (*n* = 56, 30.4%). Unexpectedly, more than a quarter of the participants could be considered workaholics based on Andreassen et al.’s (2012) classification system. This classification was used for descriptive purposes only; all inferential analyses used the continuous BWAS score.

Women and men did not differ in burnout, its two components, HP, OP, perceived stress, work addiction, or age (all *p*s ≥ 0.12, all *η*^2^ ≤ 0.013). Women reported fewer weekly working hours than men (*M* = 41.33, SD = 14.87 vs. *M* = 47.33, SD = 17.82; *F*(1, 177) = 4.00, *p* = 0.047, *η*^2^ = 0.022). Gender was nevertheless retained as a covariate on theoretical grounds (Section 2.6).

### 3.3 Correlations

Table 3 shows the Pearson correlations. Burnout correlated negatively with HP (*r* = −0.63) and positively with perceived stress (*r* = 0.65) and work addiction (*r* = 0.51; all *p*s < 0.001). OP had a weak positive correlation with burnout (*r* = 0.18, *p* = 0.013), which was confined to Exhaustion (*r* = 0.20, *p* = 0.005; Disengagement: *r* = 0.11, *p* = 0.136), and it was more strongly related to work addiction (*r* = 0.55) and perceived stress (*r* = 0.37). HP and OP were unrelated (*r* = 0.09, *p* = 0.238). Work addiction was more strongly related to Exhaustion (*r* = 0.55) than to Disengagement (*r* = 0.33). Older participants reported somewhat less burnout (*r* = −0.18, *p* = 0.013), perceived stress (*r* = −0.20), and work addiction (*r* = −0.19), whereas weekly working hours were unrelated to burnout (*r* = 0.03, *p* = 0.728).

**Table 3:** Pearson correlations between the study variables and covariates.

Variable	1	2	3	4	5	6	7	8	9
1. Burnout	–								
2. Exhaustion	0.93^***^	–							
3. Disengagement	0.86^***^	0.61^***^	–						
4. Harmonious passion	−0.63^***^	−0.55^***^	−0.59^***^	–					
5. Obsessive passion	0.18^*^	0.20^**^	0.11	0.09	–				
6. Perceived stress	0.65^***^	0.56^***^	0.62^***^	−0.49^***^	0.37^***^	–			
7. Work addiction	0.51^***^	0.55^***^	0.33^***^	−0.37^***^	0.55^***^	0.53^***^	–		
8. Age	−0.18^*^	−0.16^*^	−0.17^*^	0.12	−0.10	−0.20^**^	−0.19^*^	–	
9. Gender^a^	0.05	0.05	0.04	−0.06	0.03	0.12	0.05	−0.03	–
10. Weekly working hours	0.03	0.13	−0.13	−0.03	0.12	−0.01	0.13	−0.05	−0.15^*^

*N* = 184 (gender: *n* = 183; weekly working hours: *n* = 180; pairwise deletion). Burnout and Disengagement scores exclude MOLBI item 10; correlations of the total score with its components are part–whole correlations. ^a^0 = man, 1 = woman. ^*^p < 0.05, ^**^p < 0.01, ^***^p < 0.001 (two-tailed).

### 3.4 Hypothesis tests: hierarchical regression

Table 4 and Figure 2 present the results. The covariates entered in Step 1 explained 2.9% of the variance in burnout, *F*(3, 175) = 1.72, *p* = 0.164; only age had a significant, negative coefficient (*β* = −0.15, *p* = 0.040). Adding the four focal variables in Step 2 increased the explained variance by 54.0 percentage points, Δ*F*(4, 171) = 53.56, *p* < 0.001, to a total *R*^2^ of 0.569 (adjusted *R*^2^ = 0.551). HP (*β* = −0.39, *p* < 0.001) and perceived stress (*β* = 0.38, *p* < 0.001) had the strongest unique associations with burnout, which supports H1 and H3. Work addiction had a smaller but significant positive association (*β* = 0.17, *p* = 0.016), which supports H4. OP was not related to burnout once the other variables were controlled (*β* = −0.03, *p* = 0.691), so H2 was not supported. None of the covariates remained significant in Step 2. General dominance weights showed that HP (39.6% of the explained variance) and perceived stress (37.9%) contributed most, followed by work addiction (17.9%) and OP (3.0%); the three covariates together accounted for 1.6%.

**Table 4:** Hierarchical multiple regression of burnout on the covariates, the four focal variables, and the HP × perceived stress interaction (*N* = 179).

Predictor	*B*	SE	*β*	*t*	*p*	95% CI for *B*
Step 1: Covariates						
Age	−0.072	0.035	−0.15	−2.07	0.040	[−0.140, −0.003]
Gender (1 = woman)	0.583	0.871	0.05	0.67	0.504	[−1.136, 2.302]
Weekly working hours	0.012	0.022	0.04	0.56	0.574	[−0.030, 0.055]
*R*^2^ = 0.029, *F*(3, 175) = 1.72, *p* = 0.164						
Step 2: Focal variables added						
Age	−0.009	0.024	−0.02	−0.39	0.698	[−0.056, 0.038]
Gender (1 = woman)	−0.352	0.592	−0.03	−0.59	0.553	[−1.520, 0.816]
Weekly working hours	0.001	0.015	0.00	0.06	0.953	[−0.028, 0.030]
Harmonious passion	−0.319	0.053	−0.39	−5.97	<0.001	[−0.424, −0.213]
Obsessive passion	−0.022	0.054	−0.03	−0.40	0.691	[−0.129, 0.086]
Perceived stress	0.693	0.122	0.38	5.68	<0.001	[0.453, 0.934]
Work addiction	0.185	0.076	0.17	2.42	0.016	[0.034, 0.335]
*R*^2^ = 0.569, adjusted *R*^2^ = 0.551; Δ*R*^2^ = 0.540, Δ*F*(4, 171) = 53.56, *p* < 0.001						
Step 3: Interaction added						
HP × perceived stress	−0.013	0.015	−0.04	−0.88	0.378	[−0.043, 0.016]
*R*^2^ = 0.571; Δ*R*^2^ = 0.002, Δ*F*(1, 170) = 0.78, *p* = 0.378						

Outcome: burnout (9-item MOLBI total). *B* = unstandardized coefficient; *β* = standardized coefficient; CI = confidence interval; HP = harmonious passion. Intercepts were 22.19 (Step 1), 20.22 (Step 2), and 16.10 (Step 3). In Step 3, HP and perceived stress were mean-centered; the other coefficients changed by less than 0.01 in *β*. Five participants with missing covariate data were excluded listwise.

**Figure 2:**
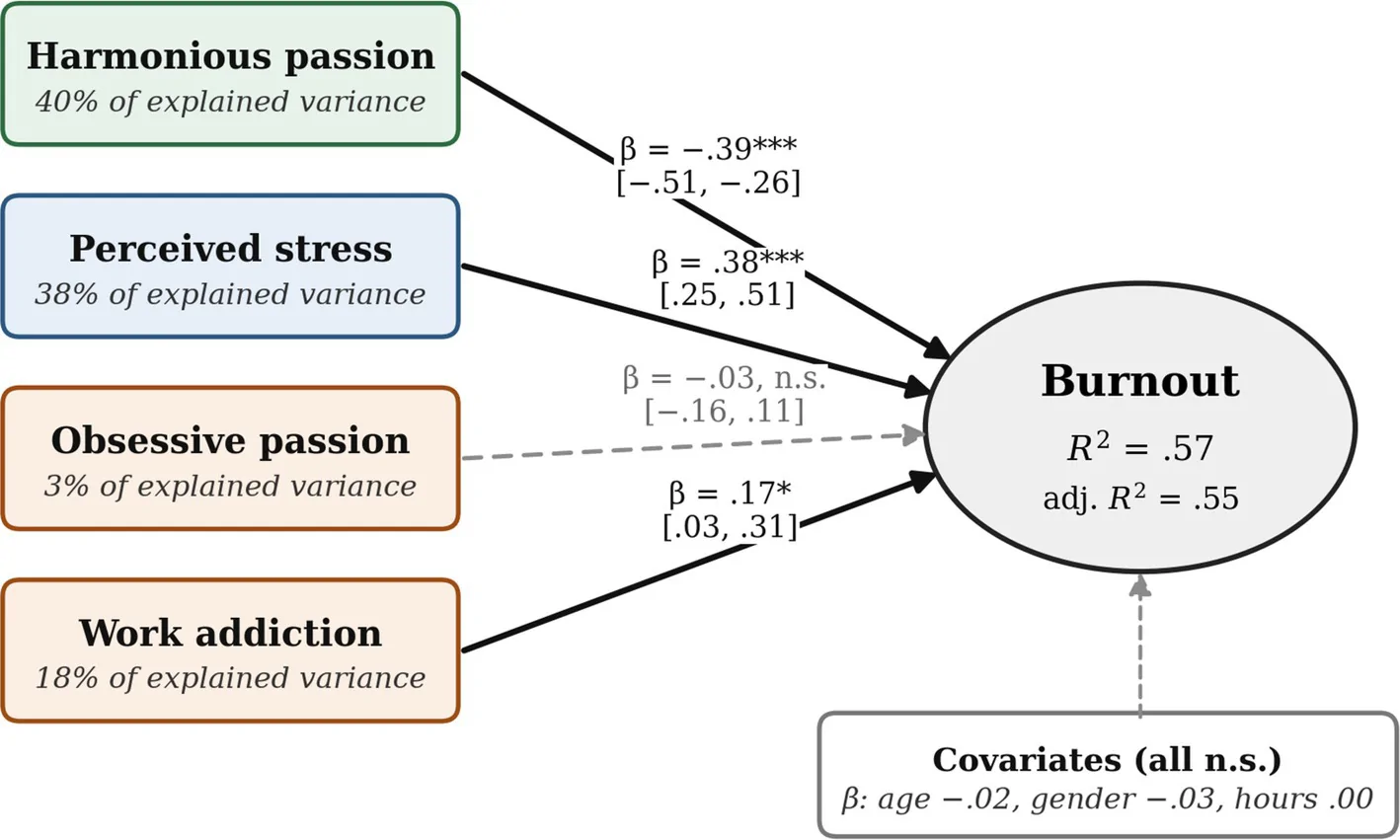
Final regression model (Step 2; *N* = 179). Values on the arrows are standardized coefficients (*β*) with 95% confidence intervals; percentages in the boxes show each variable’s share of the explained variance (general dominance weights). Solid arrows, *p* < 0.05; dashed arrows, not significant. ^*^p < 0.05, ^***^p < 0.001. Step 1 (covariates): *R*^2^ = 0.03, n.s. Step 2 (+four focal variables): Δ*R*^2^ = 0.54^***^. Step 3 (+HP × stress interaction): Δ*R*^2^ = 0.02, *β* = −0.04, n.s. (see Figure 3B).

In Step 3, the HP × perceived stress interaction was not significant (*B* = −0.013, SE = 0.015, *β* = −0.04, *p* = 0.378; Δ*R*^2^ = 0.002), so H5 was not supported. The slope of perceived stress was similar at low (−1 SD; *B* = 0.78), average (*B* = 0.70), and high (+1 SD; *B* = 0.62) levels of HP, all *p*s < 0.001 (Figure 3B).

**Figure 3:**
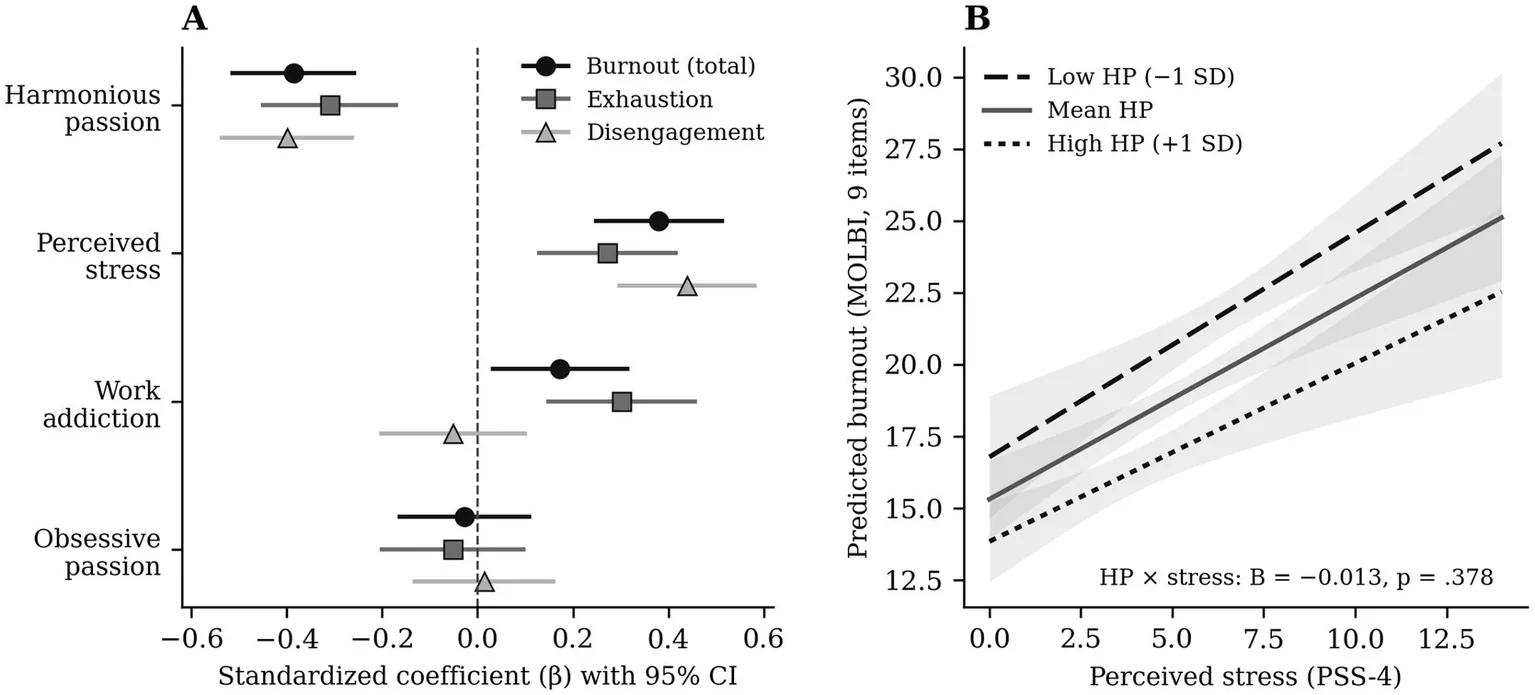
**(A)** Standardized coefficients with 95% confidence intervals for the total burnout score and its two components, from models that include the three covariates (*N* = 179). **(B)** Predicted burnout as a function of perceived stress at low (−1 SD), average, and high (+1 SD) levels of harmonious passion (Step 3 model, covariates held at their means); shaded areas are 95% confidence bands.

Regression diagnostics were satisfactory. The residuals were approximately normal (skewness = 0.21, kurtosis = 0.10; Shapiro–Wilk *p* = 0.48), there was no sign of heteroscedasticity (*p* = 0.46), no standardized residual exceeded ±3; the largest Cook’s distance was 0.08; variance inflation factors ranged from 1.04 to 2.02; and the Durbin–Watson statistic was 1.83. HC3-based tests gave the same pattern of significance (work addiction: *p* = 0.022), and the bootstrap 95% confidence intervals excluded zero for HP [−0.418, −0.208], perceived stress [0.427, 0.991], and work addiction [0.035, 0.346], but not for OP [−0.112, 0.070].

### 3.5 Robustness checks and exploratory analyses

The main findings held across all alternative specifications (Table 5). The coefficients of HP (*β* = −0.31 to −0.42) and perceived stress (*β* = 0.27–0.44) were significant in every model, whereas OP was never significant (*β* = −0.05 to 0.01). Work addiction was significant in most specifications, but its coefficient fell just short of significance with the original 10-item burnout score when the covariates were included (*β* = 0.14, *p* = 0.061) and when it was scored as the number of symptoms endorsed (*β* = 0.13, *p* = 0.051). The analyses of the two burnout components revealed a clear difference: work addiction was related to Exhaustion (*β* = 0.30, *p* < 0.001) but not to Disengagement (*β* = −0.05, *p* = 0.498), whereas HP and perceived stress were related to both (Figure 3A). The HP × stress interaction was not significant in any specification (*p*s = 0.105–0.922).

**Table 5:** Robustness checks: standardized coefficients (*β*) across alternative specifications.

Specification	*N*	*R* ^2^	HP	OP	PS	WA	HP × PS (*p*)
Main model (9-item burnout score)	179	0.569	−0.39^***^	−0.03	0.38^***^	0.17^*^	0.378
Original 10-item burnout score	179	0.553	−0.42^***^	−0.04	0.36^***^	0.14	0.488
9-item score, no covariates	184	0.572	−0.38^***^	−0.02	0.38^***^	0.18^**^	0.724
10-item score, no covariates	184	0.556	−0.41^***^	−0.04	0.36^***^	0.15^*^	0.922
Adding entrepreneurial experience and firm size	165	0.592	−0.38^***^	−0.02	0.38^***^	0.20^**^	0.493
Outcome: Exhaustion	179	0.489	−0.31^***^	−0.05	0.27^***^	0.30^***^	0.105
Outcome: Disengagement (4 items)	179	0.505	−0.40^***^	0.01	0.44^***^	−0.05	0.597
Work addiction as symptom count (0–7)	179	0.564	−0.41^***^	0.01	0.39^***^	0.13	0.522
Work addiction as cut-off classification	179	0.571	−0.41^***^	0.01	0.39^***^	0.15^**^	0.607

Unless stated otherwise, the outcome is the 9-item burnout score, and the models include age, gender, and weekly working hours. HP = harmonious passion; OP = obsessive passion; PS = perceived stress; WA = work addiction. The last column gives the *p* value of the HP × PS interaction when it is added to each model. In the main model, HC3 standard errors and percentile bootstrap confidence intervals (5,000 resamples) led to the same conclusions. ^*^p < 0.05, ^**^p < 0.01, ^***^p < 0.001.

## 4 Discussion

In this study, we examined how harmonious and obsessive passion, perceived stress, and work addiction relate to burnout in Hungarian entrepreneurs when they are considered together. Four findings stand out. First, HP and perceived stress had strong and roughly equal associations with burnout in opposite directions, and together they accounted for about three quarters of the explained variance. Second, work addiction had a smaller, independent association with burnout that was specific to exhaustion. Third, OP was only weakly correlated with burnout and had no unique association with it once work addiction and stress were considered. Fourth, we found no evidence that HP buffers the association between stress and burnout; the two variables related to burnout additively.

### 4.1 Harmonious passion and perceived stress

Our results regarding HP align with past research. For example, Castillo et al. (2017) also found that HP was a negative predictor of burnout in Spanish physical education teachers, as demonstrated via a moderate negative correlation (−0.31) and a standardized coefficient of −0.22. In our study, the connection was stronger, manifested by a strong correlation (*r* = −0.63) and a larger standardized coefficient (*β* = −0.39, even after adjustment for perceived stress, work addiction, and OP). Similarly, our results match those obtained by Fernet et al. (2014) showing that HP was inversely related to three subdomains of burnout in Canadian teachers. Furthermore, our results align with the findings of Saville et al. (2018) and Vallerand et al. (2010), who reported that HP was associated with reduced burnout in student and nurse samples. Finally, while not measuring HP, Staszczyk and Tokarz (2015) found that work enjoyment, a concept like HP, was the strongest (negative) predictor of burnout in specialists and managers of a large Polish company. Therefore, HP, reflecting internal motivation, like or even love for work and enjoyment (Vallerand et al., 2003), emerged as a correlate of lower burnout in various samples. Our results expand these works and that of De Mol et al. (2018) by showing that HP is also negatively associated with burnout in Hungarian entrepreneurs, independently of how stressed they feel and how compulsively they work.

Why might HP go together with less burnout in entrepreneurs? Self-concordance theory offers one explanation. Goals that express a person’s own interests and values are pursued with more sustained effort and yield more need satisfaction and wellbeing than goals pursued out of internal or external pressure (Sheldon and Elliot, 1999). HP is, in essence, a self-concordant way of relating to work, so entrepreneurs high in HP may invest heavily in their business without paying the same energetic price. A second explanation concerns recovery. Harmoniously passionate people can disengage from their activity when other parts of life call for attention; HP has been linked to less rumination and better recovery, whereas OP has been linked to more rumination, which in turn relates to emotional exhaustion (Donahue et al., 2012). Psychological detachment from work during off-job time is one of often-reported protective factors against exhaustion (Sonnentag and Fritz, 2015). This mechanism may be especially relevant to entrepreneurs. Unlike most employees, business owners decide for themselves when, where, and how much they work, and this autonomy is one reason why the self-employed often report less work-related stress than employees despite heavier demands (Hessels et al., 2017). The same autonomy, however, also removes external limits on work: business owners carry the financial risk of the venture, face role overload and ambiguity, and can be reached by their business at any time (Stephan, 2018). HP may help entrepreneurs use their autonomy to protect time for recovery, whereas without it, autonomy can turn into unlimited availability. It is worth adding that weekly working hours were unrelated to burnout in our sample (*r* = 0.03), which suggests that the quality of entrepreneurs’ engagement with work matters more than its quantity. These mechanisms were not measured here and should be tested directly.

Our findings regarding the association between stress and burnout align with prior research (Jamal and Baba, 2000; Lubbadeh, 2020; Membrive-Jiménez et al., 2020; Wong and Spence Laschinger, 2015), reaffirming this well-established relationship. In JD-R terms, perceived stress reflects how heavily entrepreneurs experience their demands, and its strong association with both exhaustion and disengagement is consistent with the health-impairment pathway. The negative correlation between age and perceived stress also fits the observation of Johnson et al. (2013) that older workers cope better with stressors, although age itself was unrelated to burnout once the focal variables were controlled.

### 4.2 Work addiction and obsessive passion

Work addiction was related to burnout over and above stress and passion, but only through its exhaustion component. This is broadly consistent with earlier studies. Moyer et al. (2017), using different instruments than in the current study, found a strong correlation between work addiction and burnout in American university teachers. Towch et al. (2024) also found a strong relationship between work addiction and burnout in an international sample of employees. In contrast, Nonnis et al. (2018) found that work addiction could only partially predict burnout in a large sample of Italian nurses. Similarly, Bereznowski et al. (2023) revealed that burnout was more closely related to engagement than work addiction in a Polish sample of mixed employees. Direct comparisons are limited, however, by differences in cultural context, instruments used, and, most considerably, the samples studied, which did not explicitly consist of entrepreneurs.

Schaufeli et al. (2008), investigating Dutch telecom managers, found that burnout and workaholism were strongly and positively related (*r* = 0.53), yet distinct. Our finding that work addiction was related to exhaustion but not to disengagement fits this picture. People who work compulsively deplete their energy, but they do not withdraw from their work; if anything, they remain highly involved in it. For entrepreneurs, this suggests that compulsive working threatens their energy rather than their commitment, and that exhaustion may be the earlier warning sign. Comparisons with Schaufeli et al. (2008) remain tentative, however, because the instruments used in the two inquiries were different. Also, the entrepreneurs in our study had a heterogeneous background compared to the homogenous management profile studied by Schaufeli et al. Additionally, cultural factors could influence the results. Therefore, future studies need to investigate the connection between work addiction and burnout in entrepreneurs with identical instruments and comparable, entrepreneurial contexts while accounting for cultural factors.

The high proportion of participants at risk of work addiction (30.4%) also deserves attention. It is well above the 8.3% found with the same instrument and criterion in a nationally representative sample of Norwegian employees (Andreassen et al., 2014). Differences in sampling and national context rule out firm conclusions, but the figure suggests that compulsive work patterns are common among established business owners.

OP was only weakly correlated with burnout (*r* = 0.18) and had no unique association with it once work addiction, stress, and HP were taken into account, so H2 was not supported. These findings contrast with the research by Castillo et al. (2017), Shah et al. (2020), and Fernet et al. (2014), as well as with De Mol et al. (2018) in entrepreneurs, which revealed a positive association between OP and burnout. Differences in research contexts may explain the variation in findings. For instance, while we studied Hungarian entrepreneurs across various fields, Castillo et al. and Shah et al. focused on Spanish physical education teachers and Pakistani special education teachers, respectively. Additionally, they used a different burnout measure (the Maslach Burnout Inventory). Our bivariate correlation (*r* = 0.18) was, in fact, close to those reported by Castillo et al. (0.14) and Shah et al. (0.28). In contrast, Fernet et al. examined teachers in Quebec and found a stronger association between OP and burnout than Castillo et al. and Shah et al. (2020). The main difference, therefore, lies not in the bivariate association but in what remains of it after adjustment. In our sample, OP overlapped substantially with work addiction (*r* = 0.55) and perceived stress (*r* = 0.37), and none of the studies cited above controlled for work addiction. OP is closely related to workaholism (Birkeland and Buch, 2015), and its link with exhaustion has been shown to run through rumination and poor recovery (Donahue et al., 2012). A plausible interpretation is that OP matters for burnout mainly to the extent that it translates into compulsive working and heightened strain. Cross-sectional data cannot test this mediation hypothesis, but it deserves longitudinal study.

Nevertheless, our results concerning the OP-burnout relationship appear consistent with a study examining burnout in Norwegian junior athletes. Moen et al. (2016) found that while HP significantly predicted athletic burnout, OP was not a significant predictor. Furthermore, OP was inversely and statistically significantly correlated with burnout (*r* = −0.21). However, Moen et al. used a sport-specific measure, the Athlete Burnout Questionnaire. Therefore, the similar finding that OP is not a statistically significant predictor of burnout may be coincidental because the two studies cannot be rationally compared. The message is that models for burnout are specific to a studied sample, and their generalizability to the population represented by the studied sample can be assumed only after several replications under similar conditions (job activity, instrument, cross-cultural context, etc.).

### 4.3 No evidence of buffering

Contrary to H5, HP did not weaken the association between perceived stress and burnout, and this held for both components of burnout. The idea that HP counteracts stress therefore finds no support in our data; what we observed were two independent associations of opposite sign. This additive pattern fits COR theory (Hobfoll et al., 2018) at least as well as the JD-R buffering hypothesis. HP may be linked to lower burnout because it is a resource in its own right, and perhaps because it goes together with less threatening appraisals of demands (Lazarus and Folkman, 1984; HP and perceived stress correlated at *r* = −0.49), rather than because it changes how stress translates into burnout. Two caveats apply. First, interaction effects in field studies are typically small and hard to detect (McClelland and Judd, 1993); with our sample, we could reliably detect only interactions that explained about 2% of additional variance, so a weaker buffering effect cannot be ruled out. Second, buffering by HP has been observed in entrepreneurs for other outcomes: Stroe et al. (2020) found that HP dampened the link between fear of failure and negative affect, a more immediate emotional reaction than burnout. Whether HP buffers acute reactions but not chronic strain is a question for future research.

### 4.4 Theoretical and practical implications

The study contributes to theory in three ways. First, it supports treating HP as a personal resource within the JD-R framework (Xanthopoulou et al., 2007) and shows that, among entrepreneurs, this resource relates to burnout additively rather than by buffering stress. Second, it refines the dualistic model of passion for the entrepreneurial context: the burnout risk usually attributed to OP appears to be shared with compulsive working and strain, so studies that examine OP without work addiction may overstate its independent role. Third, it shows that work addiction and HP relate to different facets of burnout. Work addiction was tied to exhaustion only, whereas HP and stress were tied to both exhaustion and disengagement. Research on entrepreneurial burnout should therefore examine its components as well as the total score.

Several practical implications follow, although they should be read in light of the cross-sectional design. Programs for entrepreneurs could aim to strengthen HP, for example, by helping business owners pursue goals that reflect their own values and interests, keep their business compatible with other life domains, and protect time for recovery. Like employees, entrepreneurs can also benefit from stress management. Because almost one in three participants met the criterion for being at risk of work addiction, screening for compulsive work patterns (e.g., with the BWAS) could help identify those most at risk of exhaustion. Chambers of commerce and entrepreneurs’ associations, which helped us to reach our participants, are well placed to offer such support.

### 4.5 Limitations and future research

This study has limitations that must be considered when interpreting the findings. First, the cross-sectional design prevents any causal conclusions. The associations could run in either direction; burnout may, for example, erode HP or intensify stress appraisals. Second, the volunteer sample may be biased and differ from those who did not or would not volunteer for this type of research. Third, the sample’s average age suggests an older demographic, which may not fully reflect younger entrepreneurs’ burnout experiences. Additionally, with only one-quarter of the participants being women, the findings may not be generalized to a more gender-balanced population. Fourth, the cultural homogeneity of the sample studied prevents the generalization of the findings to other cultures. Fifth, recruiting and examining entrepreneurs from any field might yield different results than studying entrepreneurs in a particular field or sectors. Sixth, like all online research, there was no control over respondents’ matching the participation criteria.

Seventh, all variables were measured with self-reports at a single time point, so the associations may be inflated by common method variance (Podsakoff et al., 2003). We reduced this risk procedurally by guaranteeing anonymity and by using established scales with different response formats. In a Harman single-factor test, the first unrotated factor accounted for only 26.9% of the variance in the 32 scale items (principal axis factoring), but this test is insensitive and cannot rule out method bias (Podsakoff et al., 2012). Future studies should separate the measurement of predictors and outcomes in time (e.g., by several weeks), combine self-reports with other sources, such as ratings by spouses or business partners and objective indicators of workload or firm performance, and continue to guarantee respondents’ anonymity.

Eighth, one reverse-keyed MOLBI item did not work as intended and was excluded, and the Disengagement subscale was only moderately reliable even without it (*α* = 0.74). Reverse-keyed items are a known weak point of OLBI-type instruments (Mészáros et al., 2020), and replications should check whether this item functions differently among business owners. Ninth, the BWAS cut-off identifies people at risk of work addiction and is not a clinical diagnosis. Tenth, as noted above, our power to detect small interaction effects was limited. Finally, relevant variables such as neuroticism, the financial situation of the firm, and social support were not measured and could account for part of the observed associations.

Future research should use longitudinal or diary designs to establish the temporal order of passion, stress, compulsive working, and burnout; test whether rumination and psychological detachment explain the associations of HP and OP with exhaustion; and compare entrepreneurs with employees in the same study to identify what is specific to entrepreneurship and also what is specific to employees.

## 5 Conclusion

Our study offers new insights into how passion, perceived stress, and work addiction relate to burnout in entrepreneurs. When these variables were examined together, perceived stress and HP emerged as strong, opposing correlates of burnout; work addiction added a smaller association that was specific to exhaustion; and OP had no independent association. HP did not buffer the association between stress and burnout; instead, it behaved as an additive personal resource. Together, the four variables accounted for 54% of the variance in burnout beyond age, gender, and working hours. These findings underline the value of fostering HP, which aligns with intrinsic motivation and job enjoyment, and of addressing compulsive work patterns when promoting entrepreneurial wellbeing. Because the data are cross-sectional, further research is needed to refine these relationships, particularly by using longitudinal designs and consistent measures and by considering cultural differences.

## Data Availability

The raw data supporting the conclusions of this article will be made available by the authors, without undue reservation.
